# Supplementary material for: Exploring Community-Based Options for Reducing Youth Crime
Source: Int J Environ Res Public Health. 2021 May 12;18(10):5097. doi: 10.3390/ijerph18105097 (PMC8150417; doi:10.3390/ijerph18105097)
Supplement: Supplementary file 1 [file ijerph-18-05097-s001.zip › ijerph-1205332-supplementary.pdf]

Resp. No.

Youth Crime Survey

We are conducting a study on youth crime and would like you to consider the following scenario:

In the Armidale area in 2013, 249 crimes were committed by 14-17 year olds.

Your local government is concerned at the rates of youth crime and antisocial behaviour (e.g. drug & alcohol use, vandalism, assault).

You are attending a regional forum which is being held to gather community responses to different ways of dealing with youth crime. The forum would like to know which of the two following options to reduce youth crime you would be prepared to pay for:

1) Greater Police Presence
An increase in police numbers and a greater police presence on local streets. The aim is to prevent youth crime and apprehend those who commit crime.

2) BackTrack
A community-based program, which directs at risk youth into activities that enable them to contribute to their community. The aim of BackTrack is to assist these young people to develop employment opportunities, improve their school attendance, improve their health and wellbeing and reduce their involvement in substance abuse and crime.

The Survey
First, we will present you with 9 choices. For each choice you will be asked to decide which option is the best. You will need to think about:

1. How much each option reduces youth crime.

2. How much each option costs per household. This is the amount that your household would have to pay each year to address youth crime in addition to your usual taxes that pay for police.

After the 9 choice questions, we ask some general questions about your choices and about you (gender, age, education, etc.). Such information enables us to compare the choices of people of different ages or from different backgrounds.

Please remember:

Your answers are totally anonymous

You can stop at any time.

1. Tick one box in *Your Choice*

	BackTrack	***Greater Police Presence***
	Achieves 20% less crime	Achieves 30% less crime
	Costs $60 per year	Costs $120 per year
Your Choice	☐	☐

2. Tick one box in *Your Choice*

	BackTrack	***Greater Police Presence***
	Achieves 20% less crime	Achieves 10% less crime
	Costs $120 per year	Costs $60 per year
Your Choice	☐	☐

3. Tick one box in *Your Choice*

	BackTrack	***Greater Police Presence***
	Achieves 10% less crime	Achieves 30% less crime
	Costs $30 per year	Costs $60 per year
Your Choice	☐	☐

4. Tick one box in *Your Choice*

	BackTrack	***Greater Police Presence***
	Achieves 10% less crime	Achieves 20% less crime
	Costs $60 per year	Costs $120 per year
Your Choice	☐	☐

5. Tick one box in *Your Choice*

	BackTrack	***Greater Police Presence***
	Achieves 10% less crime	Achieves 20% less crime
	Costs $30 per year	Costs $60 per year
Your Choice	☐	☐

6. Tick one box in *Your Choice*

	BackTrack	***Greater Police Presence***
	Achieves 10% less crime	Achieves 30% less crime
	Costs $60 per year	Costs $120 per year
Your Choice	☐	☐

7. Tick one box in *Your Choice*

	BackTrack	***Greater Police Presence***
	Achieves 30% less crime	Achieves 10% less crime
	Costs $120 per year	Costs $30 per year
Your Choice	☐	☐

8.Tick one box in *Your Choice*

	BackTrack	***Greater Police Presence***
	Achieves 30% less crime	Achieves 10% less crime
	Costs $60 per year	Costs $30 per year
Your Choice	☐	☐

9. Tick one box in *Your Choice*

	BackTrack	***Greater Police Presence***
	Achieves 20% less crime	Achieves 30% less crime
	Costs $30 per year	Costs $120 per year
Your Choice	☐	☐

Questions about You

Please answer each question by placing a tick in one box

1. **Unlike greater police presence which has a specific focus on trying to reduce crime rates, BackTrack provides other benefits for young people, such as reduced substance use, better physical and mental health, greater confidence, improved school attendance and completion, and being more job ready.**

   Given the value of these added benefits, how much more would you be willing to pay to have BackTrack in your community:

   Nothing ☐
   Twice as much ☐
   Five times as much ☐
   Ten times as much ☐

   Other ______________________

2. **What is your gender?**

   ☐ Male
   ☐ Female

3. **What is your age?**

   ☐ 18-29
   ☐ 30-49
   ☐ 50-69
   ☐ 70 or older

4. **Are you employed?**

- ☐ Full Time
- ☐ Part Time/Casual
- ☐ Unemployed
- ☐ Not in the Workforce

5. **What is your highest level of education?**

- ☐ Less than Year 12
- ☐ Year 12
- ☐ Apprenticeship or Diploma
- ☐ Bachelor Degree
- ☐ Postgraduate

6. **What is your annual household income?**

- ☐ Less than Year $20,000
- ☐ $20,000-$39,000
- ☐ $40,000-$59,000
- ☐ $60,000-$79,000
- ☐ $80,000 or more
- ☐ Prefer not to say

7. **What is your postcode of usual residence** ______________________

Thank you very much for participating in this survey

The results will assist in supporting new programs to decrease levels of crime and support young people and communities in NSW
